# Novel Metaphors Comprehension in a Child with High-Functioning Autism Spectrum Disorder: A Study on Assessment and Treatment

**DOI:** 10.3389/fpsyg.2016.02004

**Published:** 2017-01-04

**Authors:** Sergio Melogno, Maria A. Pinto, Margherita Orsolini

**Affiliations:** Dipartimento di Psicologia dei Processi di Sviluppo e Socializzazione, “Sapienza,” Università di RomaRome, Italy

**Keywords:** novel metaphors, comprehension, autism spectrum disorder, high functioning, assessment, treatment

## Abstract

Until the first decade of the current millennium, the literature on metaphor comprehension highlighted typical difficulties in children with high-functioning Autism Spectrum Disorder (ASD). More recently, some scholars have devised special programs for enhancing the capability of understanding metaphors in these children. This article presents a case study based on a treatment aiming at enhancing novel metaphor comprehension in a high-functioning child with ASD. M.M., a pseudoacronym for an 8;10 year-old boy, diagnosed with high-functioning ASD, was first assessed with a metaphor comprehension test. This testing (at time T0) highlighted a rigid refusal of metaphors and a marked tendency toward literal interpretation. A baseline treatment (8 sessions of 45–60 min each, twice a week) was implemented, based on a series of recognition, denomination and emotion comprehension activities. M.M.'s metaphor comprehension was assessed a second time (T1), followed by the experimental treatment (same duration and frequency as the first one), specifically focused on metaphor comprehension. Finally, a third assessment of metaphor comprehension took place (T2), followed by a last assessment 4 months later (follow-up, T3). The comparison between the performances at the metaphor comprehension test across the four assessments, from T0 to T3, showed that the baseline treatment produced no effect at all, whereas a significant improvement appeared at T2, just after the experimental treatment, later confirmed at the follow up. Both quantitative and qualitative results showed an evident improvement in the way M.M. handled the semantic issues posed by the metaphors of the test, in line with the strategies he was taught during the treatment.

## Introduction

Metaphor comprehension difficulties in high-functioning children with Autism Spectrum Disorder (ASD, henceforth) are well documented (Happé, 1993, 1995; Norbury, 2005; Rundblad and Annaz, 2010; Melogno et al., 2012a,c; Kasirer and Mashal, 2014; Vulchanova et al., 2015). Faced with metaphors, children with ASD tend to remain firmly anchored to literal interpretations. For instance, if one of two children say “Autumn leaves are butterflies,” the listener might interpret the relationship between the two terms (*X* = autumn leaves, *Y* = butterflies) literally, i.e., as an identity, and fails to understand the speaker's intended meaning. In this case, he/she can only reject the sentence because autumn leaves are clearly *not* butterflies. However, if the listener realizes that what the speaker means is “Autumn leaves *are like* butterflies,” he/she can compare the two terms and look for a shared property, in this case, the movement or the shape, the color, etc., and come to a plausible interpretation of the metaphor.

In developmental literature, metaphors like the one above are called *sensory* (Winner, 1998), because they combine terms pertaining to the same physical sphere, in which the link between the two parts of the metaphor relies on functional or perceptual properties (color, shape, etc.). Other metaphors are called *physico-psychological* (Winner, 1998) because the two terms that compose them pertain to the physical and the psychological domain (e.g., “Juliet is the sun”). Another relevant distinction is between dead and novel metaphors, the former being those we conventionally use (e.g., “He is a snake”), and the latter being created on the spot (e.g.,“Autumn leaves are butterflies”).

Based on empirical evidence, 6–7 year olds typically developing children are already able to guess that metaphors are a deliberate means to communicate and not merely a mistake or a lie (Demorest et al., 1983). The first signs of metaphorical understanding can even be found at the age of 4 (Keil, 1986; Gentner, 1988), although this ability is gradually refined later on (Vosniadou, 1987; Declercq et al., 2010).

Research on metaphor comprehension has found lower performance in high functioning children with ASD than in neurotypical children (Happé, 1993, 1995; Dennis et al., 2001; Martin and McDonald, 2004; Norbury, 2005; Rundblad and Annaz, 2010), particularly in a subgroup characterized by language difficulties (Norbury, 2005; Gernsbacher and Pripas-Kapit, 2012). How to explain impaired metaphor comprehension in children with ASD is still an open issue. Some scholars (Happé, 1993, 1995) claim that a deficit in the theory of mind would undermine the comprehension of what the speaker means (X *is like* Y) beyond what the speaker says (X *is* Y). However, Norbury (2005) showed that theory of mind is a “necessary but not sufficient” factor to metaphor comprehension and that specific language competences such as semantic knowledge are also necessary. Another possible explanation comes from the tendency (Weak Central Coherence) shown by people with ASD to process information locally rather than globally. This could have negative consequences on the ability to use sentence context to disambiguate meaning (Rundblad and Annaz, 2010). The efficiency of executive functions is another factor that may account for the difficulties encountered by children with ASD (Mashal and Kasirer, 2011; Kasirer and Mashal, 2014).

Recent research based on experimental and case studies has raised the question of whether metaphor comprehension can be enhanced in children with ASD (Mashal and Kasirer, 2011; Persicke et al., 2012; Melogno et al., 2012b). Adapting a procedure used in brain lesions rehabilitation (Lundgren et al., 2006), Mashal and Kasirer (2011) created *thinking maps* to train children in comparing the two terms of a metaphor. In a similar vein, Persicke et al. (2012) devised a procedure to help children with ASD in analyzing metaphors embedded in stories. Step by step, children were guided toward rejecting irrelevant semantic features and abstracting the relevant ones. The encouraging results of these studies suggest that teaching children with ASD how to compare terms in a metaphor can enhance metaphor comprehension. On the other hand, research on this type of intervention could illuminate the role of the many factors invoked so far to explain difficulties in metaphor comprehension in children with ASD. For instance, a training focused on semantic knowledge could test the relevance of this factor, as claimed by Norbury (2005).

The present case study extends the training method set out by Mashal and Kasirer (2011) by including renaming exercises aimed at enhancing semantic flexibility and adult-child interaction aimed at stimulating metalinguistic explanations.

## Background

M.M. is a high functioning child with ASD (American Psychiatric Association, 2013) who came to consultation in a clinical center on developmental disabilities when he was 8 years and 10 months and was diagnosed with Asperger Syndrome based on DSM IV-TR (American Psychiatric Association, 2000) criteria. The procedure involved the Autism Diagnostic Observation Schedule (ADOS; Lord et al., 2000), the Krug Asperger Disorder Index (KADI; Krug and Arick, 2003) and the Social Responsiveness Scale (Constantino and Gruber, 2002).

M.M. had fluent language but relevant difficulties in managing interactions. Namely, he tended to direct conversation toward his preferred topics on a particularly erudite mode. His parents also reported that M.M. took things too literally and didn't understand the real meaning of the conversation. M.M.'s rigid interpretation of language usages were paralleled by other forms of rigidity in behavior, at home and school, that could be observed in everyday ritualistic sequences. M.M.'s intellectual level was within normal range (Total IQ = 110), as emerged from clinical assessment (WISC III, Wechsler, 1991)^1^, with a Verbal IQ of 115 and a Performance IQ of 103. Performances within norms were expected in grammar and in lexical competencies, which was confirmed by the Test of Reception of Grammar (TROG-2; Bishop, 2003; standard score: 107) and by the Peabody Picture Vocabulary Test (PPVT; Dunn and Dunn, 1981; standard score: 115). On the other hand, based on the diagnosis, a drop in metaphor comprehension was expected, which was also confirmed by a subtest assessing figurative language (APL Medea; Lorusso, 2007). In the first part of this subtest (“verbal metaphors”), the child has to listen to either a metaphorical sentence (e.g.,“Mark is a lion”), or an idiomatic expression and must explain its meaning; in the second part (“visual metaphors”), the child has to listen to an idiomatic expression (e.g.,“You have your head in the clouds”), and must indicate the picture that matches the appropriate meaning choosing among four possibilities. Seven times out of 8, M.M. gave a literal interpretation.

In the Theory of mind and Affect recognition tests of NEPSY II (Developmental Neuropsychological Assessment; Korkman et al., 2007), difficulties in mentalization and emotion recognition were expected, which were confirmed by low performance in the first test (scaled score: 6) and impaired performance in the other (scaled score: 4).

## Discussion

### The research design

After approval from the Ethics Committee of the Dipartimento di Pediatria e Neuropsichiatria Infantile (“Sapienza” University of Rome; Department of Pediatrics and Child Neuropsychiatry, “Sapienza” Univ. of Rome) we asked an informed consent to M.M.'s parents who accepted to have their child involved in a treatment. M.M. himself consented verbally.

After clinical diagnosis, M.M.'s metaphor comprehension was first assessed with a specific test (Time 0). Then, M.M. was engaged in a baseline treatment focused on Theory of Mind (8 sessions of 45–60 min each, twice a week) and assessed a second time with a metaphor comprehension test (T1). Then an experimental treatment followed (same duration and frequency as the baseline one) targeting metaphor comprehension. Finally, a third assessment of metaphor comprehension took place (T2), followed by a follow-up assessment 4 months later (follow-up, T3).

### Testing the treatment effects: metaphor comprehension assessment

Considering the M.M.'s difficulties in non-literal understanding, we chose the Junior Metaphor Comprehension Test (Henceforth, Jnr MCT, Pinto et al., 2008), an Italian instrument validated for 4–6-year-old children. The test assesses the ability to explain the meaning of 12 metaphors included in sentences, and 13 metaphors contextualized in four stories. Nearly all of these metaphors are “sensory,” e.g.,“The moon is a light bulb,” where “moon” pertains to the domain of “celestial bodies” and “light bulb” to that of “electric devices.”

The coding system is based on a three-step scale.

A score of 0 is assigned when the child declares he/she just does'nt know (*elusion*), or refuses the possibility of using words metaphorically *(refusal*), or interprets the metaphor literally (*literal interpretation*).

A score of 1 is assigned when a semantic feature common to both terms of the metaphor is identified, i.e., a relevant common ground, based on functional or perceptual characteristics (*identification of a relevant semantic ground*).

A score of 2 is assigned when the explanation considers both differences and resemblances between the two terms of the metaphor (*identification of an elaborated ground)*. The maximum score of the Jnr MCT is 50.

Jnr MCT has high reliability, as measured by Cronbach'alpha (0.860), high test-retest correlations (r–tt:0.848), and high interrater agreement, as measured by Cohen's K (0.73 for the 5 year olds and 0.74 for the 6 year olds).

In the follow-up assessment we administered both the Jnr MCT and the APL-Medea's subtest assessing figurative language^2^.

### Baseline and experimental training

The baseline training involved the child in watching videoclips together with the adult, and in a subsequent discussion that stimulated Theory of Mind. The child was asked to identify the visual cues of a character's emotion, name the targeted emotion, and infer the characters' mental states. The experimental training was based on the following hypotheses: (a) novel metaphors generate a cognitive conflict, as the metaphorical meaning conflicts with the literal one; (b) a child with ASD can approach such a conflict as a problem-solving activity, by analyzing meanings on explicit grounds; (c) inhibiting a literal interpretation, switching from one semantic feature to another, considering a whole discourse context to comprehend a sentence, are all processes that can be taught to children with ASD. Based on these hypotheses, three main activities were implemented. The first activity, “X *is like* Y heuristic” (strategy 1), consisted of exercises training the child to inhibit a literal interpretation, change a metaphor into the corresponding simile (“X *is like* Y”) and use thinking maps to search for semantic similarities between X and Y (strategy 2, *Comparative strategy*. Table 1). In the second activity (Table 2), the child was asked to use unconventional labels to rename objects or images. When renaming concerned associates (flour re-named as bread) the adult provided examples of metaphorical labels (flour re-named as snow).

**Table 1:** **Examples of activities with the *X is like Y* heuristic**.

**Example of modeling**	
The adult read the sentence:“Skyscrapers are the city's giraffes”	
Strategy 1: “*Is like* strategy” 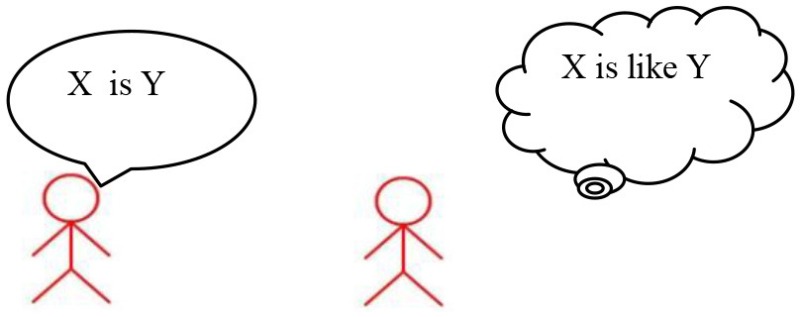	If someone says “skyscrapers are the city's giraffes,” people who listen to that sentence know that skyscrapers simply cannot be giraffes. Skyscrapers are buildings and giraffes are animals. But, to understand what is meant by this sentence I may use my first strategy with cards. I will replace X with Y and add “is like.” Then, the sentence becomes skyscrapers **are** ***like*** **giraffes**.
Strategy 2: *Comparative strategy* 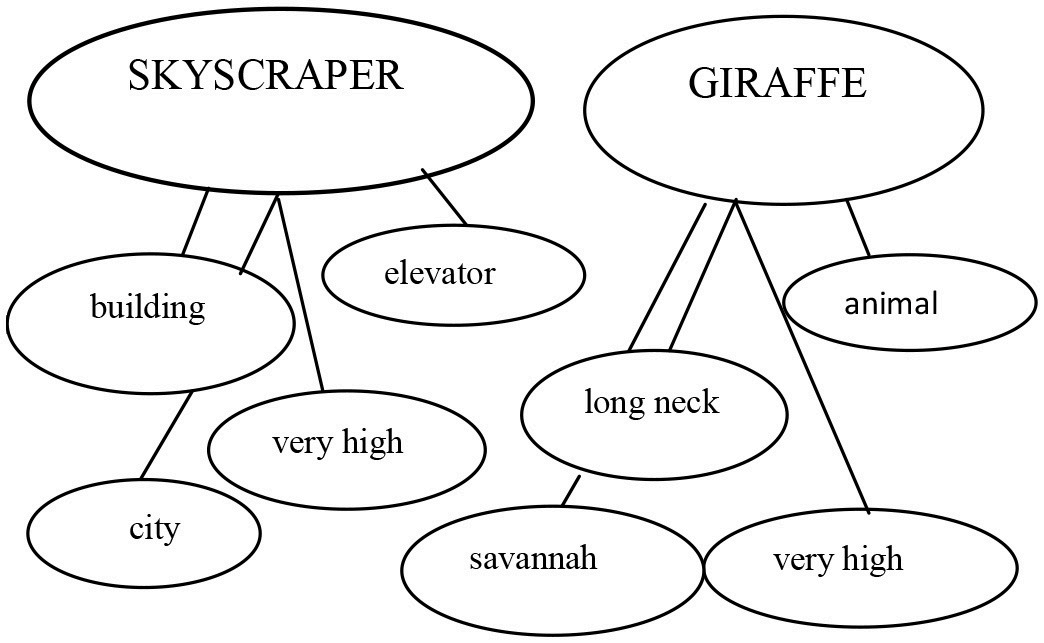	Now, I will use my second strategy. I'll be thinking of the characteristics of the skyscrapers and write them down here in my thinking map. Then, I'll write that skyscrapers are buildings, that they are very high, they have elevators, etc. Now, it's giraffes' turn. I'll be thinking of the characteristics of the giraffes and write them down in my map. Giraffes are animals, they are very high, have a long neck, they live in savannahs, etc. Right. Now, I'll see which characteristics are appropriate for *both* skyscrapers and giraffes. For instance, “building” is appropriate for skyscraper but has nothing to do with giraffes, then I'll reject it…….”Very high” goes for both, then, I'll accept it as an appropriate characteristic. Skyscrapers and giraffes are both very high.
Metalinguistic reflection	Then, we may state that the guy who said “skyscrapers are giraffes” actually meant that skyscrapers are the highest buildings in a city just as giraffes are the highest animals in a savannah. The word “giraffe” replaces another one, in this case “very high,” because it expresses the meaning of being very high.
Example of joint construction	
Strategy 1: “*Is like* strategy” 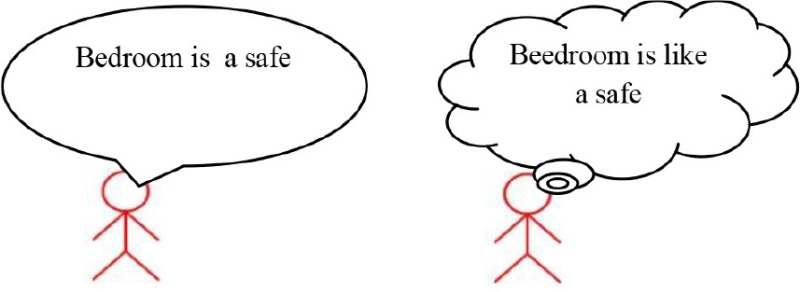	Adult: “What does this sentence mean: *Bedroom is a safe* ?” M.M. “Bedroom is like a safe” Adult: “Ok, shall we use strategy 1 and write down the new sentence in the bubble ?”
Strategy 2: *Comparative strategy* 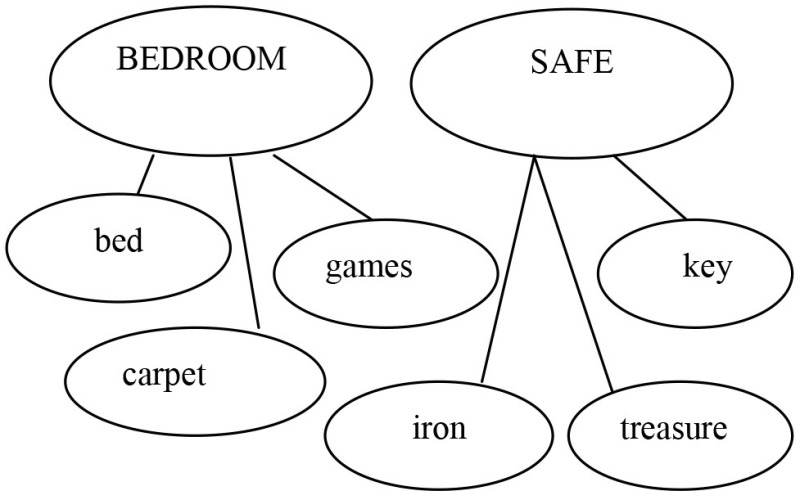	Adult: “Now we can say all we know about safes.” M.M.: “It can be iron made, and there can be money or jewelery inside. You can open it with a key or a code. Pharaons used to have the room of treasure, like Sethi in the Kings' Valley.” The adult invited M.M. to write down these features in the bubbles of the thinking map. M.M. wrote IRON, KEY, CODE, MONEY, TREASURE for the word *Safe* and BED, CARPET, GAMES for the word *Bedroom*. MM. was asked to select characteristics that can be common to *Bedroom* and *Safe*. M.M. selected KEY and discarded every other feature except TREASURE. M.M.: “A bedroom can have treasures.” Adult: “Treasures like those of Sethi”? M.M.: “No, perhaps a precious collection of shells, marbles or bus tickets. Nobody has to touch them, as with a safe.”
Metalinguistic reflection	Adult and M.M. came to the conclusion that a bedroom is a suitable place where to keep precious objects.
**Example of autonomous activity**	
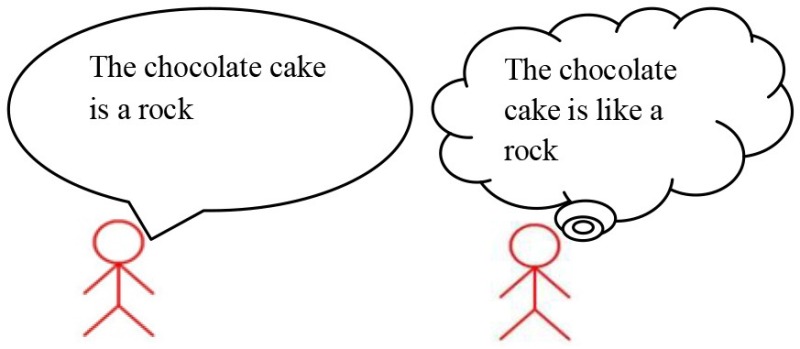	“The chocolate cake is a rock” The child had to insert the sentence in the bubbles using strategy 1 (written exercise) like in the example in the left column.
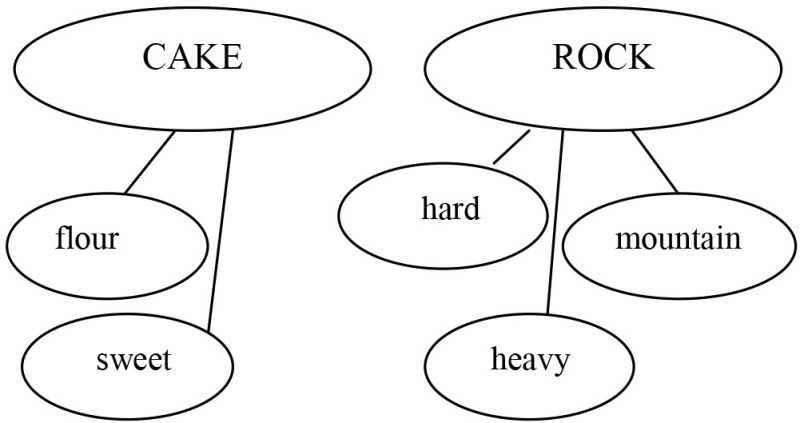	The child had to build the thinking map with CAKE and ROCK (written exercise) like in the example in the left column.
Metalinguistic reflection (oral discussion)	Adult: “What does it mean, then, that the chocolate cake is a rock?” M.M.: “Well, it means that the cake is dried, tough, you really cannot eat it.”

**Table 2:** **Examples of renaming and story-matching activities**.

**Renaming**	
The adult showed M.M. a signaling disk (X) and asked him to rename it. 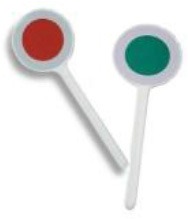	M.M.: “A lollipop.” If the child had renamed the object in a non-metaphorical way (e.g., “flour” renamed as “bread,” or “bag”), the adult might have proposed an appropriate solution attributable to *another child*. “You know, another child told me so: “flour”-“snow.”
The adult asked M.M. to justify the new label (e.g., “lollipop”) chosen for renaming.	M.M: “It has the same round shape. The color can be red as a strawberry-flavored lollipop, or green as mint-flavored lollipop.”
The adult asked M.M to select or draw a picture that matched the label chosen for renaming.	M.M chose the image that matched the new label. 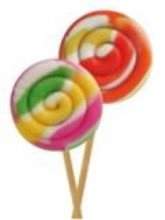
The adult asked M.M to phrase the association between the object (X) and the label he chose (Y) as a “X is Y” type of metaphor.	M.M.: “The signaling disk is a mint-and-strawberry flavored lollipop.”
**Story-matching**	
The adult told M.M. the following story: “At school, the pupils are lining up to enter into the classroom. Alice is the smallest child of the group….” Which one of the following endings of the story is the most appropriate, “Alice is a ladybug” or “Alice is a giraffe” ?	M.M. chose the appropriate alternative. (“Alice is a ladybug”).

The third activity were story-matching exercises where the child had to listen to short stories and then choose between two different metaphorical sentences to conclude the story (Table 2). The experimental training was implemented in two different phases, in 8 sessions of 45–60 min, twice a week, using novel sensory metaphors. Each session of the first phase (2 weeks) included one *X is like Y* exercise, explained and modeled by the therapist (SM), then one exercise of joint construction of metaphorical meaning, and two exercises of renaming. The *X is like Y* heuristic was consolidated through joint construction and autonomous activities in phase 2, which also included story-matching exercises.

In the whole, the child was involved in 16 “X is like Y,” 48 renaming and 12 story-matching exercises. None of the metaphors used for the assessment was included in the exercises.

## Results

Table 3 (A,B) shows the changes in M.M.'s performance at the Jnr MCT as a function of the different assessment phases.

**Table 3:** Raw (subparts and total) and standardized scores^*^ at each testing time (A); Frequencies and percentages of different levels of answers at each testing time (B).

	**Time 0**		**Time 1**		**Time 2**		**Time 3**	
	**Raw scores**	***Z***	**Raw scores**	***Z***	**Raw scores**	***Z***	**Raw scores**	***Z***
**(A)**								
Met-Sentences	7		7		18		21	
Met-Stories	3		4		21		22	
Total	10	−2.13	11	−1.95	39	3.14	43	3.87
**(B)**								
Level 0	15 (60%)		14 (56%)		2 (8%)		2 (8%)	
Level 1	10 (40%)		11 (44%)		7 (28%)		3 (12%)	
Level 2	0		0		16 (64%)		20 (80%)	

* *Standardized scores are computed on the total correct answers*.

At T0, M.M.'s performance was lower than that of a 6-year-old typically developing child. In particular, 0 level answers were prevalent (60%), and mainly consisted of refusals (“It's not true,” “It can't be,” “It doesn't exist”). The other 40% was entirely at level 1 (relevant basic ground between the two metaphor terms).

At T1, after the baseline treatment, the distribution of percentages of answers by levels was nearly identical to that obtained at T0. At T2, after the experimental training, M.M.'s 0 level answers nearly disappeared, level 2 answers (elaborated ground between the two terms of the metaphor) were produced for the first time and prevailed on level 1 answers. At T3, the total Jnr MCT's score was further increased and higher compared to 6-year old typically developing children. The massive decrease of level 0 answers was confirmed, and a further increase of level 2 answers was observed.

M.M.'s answers at the item “The scarf of the sky is colorful” across the four testing times significantly show his qualitative improvements in processing the metaphors of the test. At T0 and at T1, the child stated unconditionally: “It's not true! The sky cannot have scarves!” (*refusal*). After the treatment, at T2, the answer was more complex and accurate. “I don't have a clue of what it might be, it might be the clouds or the mountains……it's the rainbow!.” For the first time, M.M. envisaged more than one interpretation, and moved from a negative certitude (X *cannot be* Y) toward a plurality of possible meanings (X *might be* Y *or* Z), and eventually came to a plausible metaphorical interpretation (X *can be* Y). At T3, the child's answer was: “The milky way is the colorful scarf of the sky…because planets are made of different materials and each has a color. For example, Mars'colour is red…Uranus'colour is blue…and the Earth's color is a mixture between blue, which is the sea, and green…the grass.…on the soil, which is brown.” M.M.'s evolution is very evident. He did not give up his typical erudite mode; rather, he used it for *explaining* linguistic meanings (“because”) instead of *asserting* things. In this case, M.M. provided an explanation of a semantic association between the “colorful scarf of the sky” and the “milky way,” which is itself a novel metaphor.

**Figure 1:**
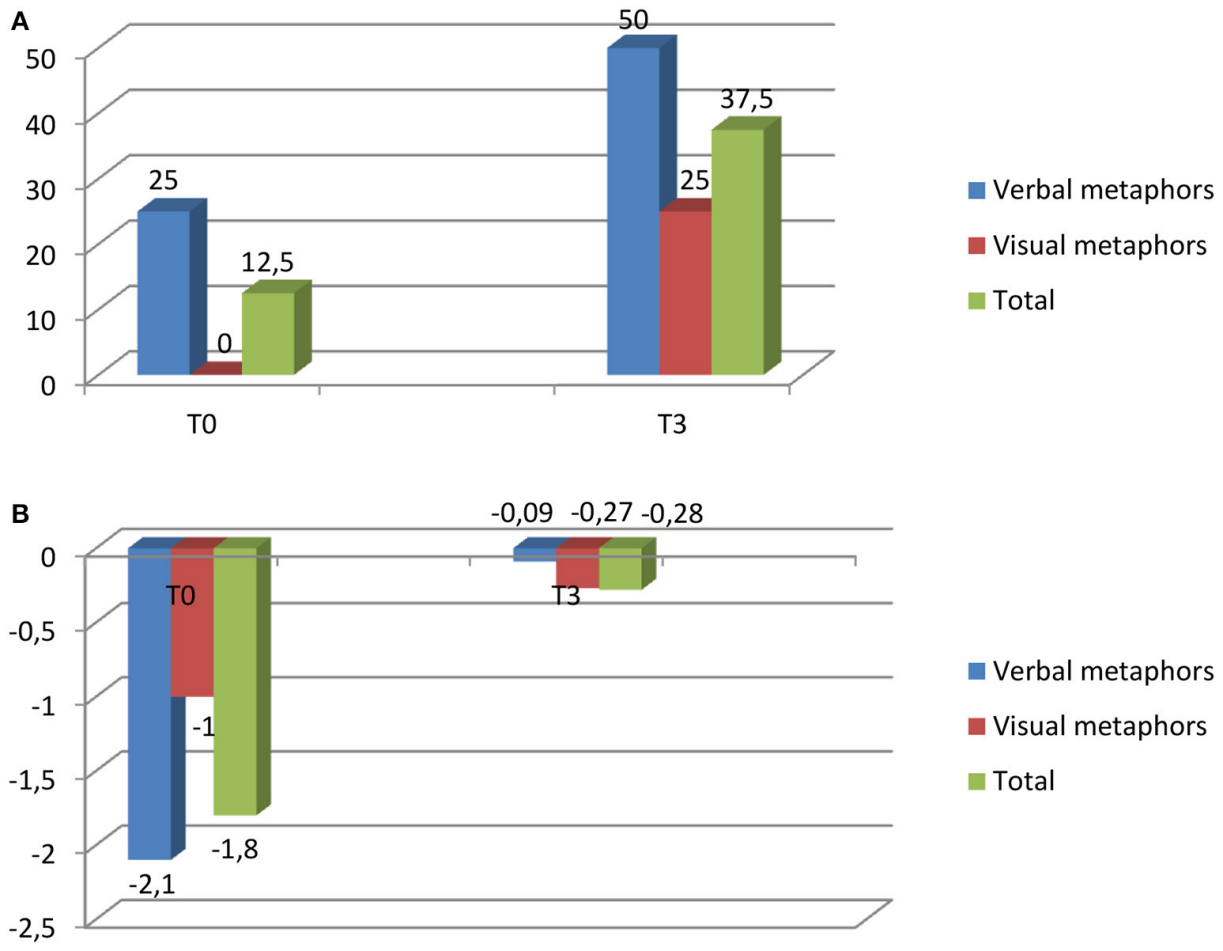
**Results at the APL test. (A)** Percentage of correct answers for verbal and visual metaphors at the APL test at T0 and T3^3^. **(B)**^4^ Standardized scores of correct answers for verbal and visual metaphors at the APL test at T0 and T3.

To test whether there were significant differences between total scores at different testing times (T0–T3), we calculated the standard error measurement. The results showed only one significant difference, that between T1 and T2 (S_*e*_: 4.6764, significant at *p* < 0.05 level). This confirms that the only significant improvement in M.M.'s performance at the Jnr MCT took place after the experimental training. In addition to the Jnr MCT, in the follow-up assessment the APL- Medea, already used in the diagnosis, was also administered. As shown in Figure 1, M.M. remarkably improved both in verbal and visual metaphors. The results were within norms.

## Concluding remarks

From the initial diagnosis to the follow-up assessment M.M. moved from 0 to 1 correct answer for visual metaphors and from one level 1 score to two level 2 scores for verbal metaphors.

The aim of this study was to analyze the effectiveness of an experimental treatment on the comprehension of novel metaphors in M.M., a child with ASD.

Enhancing M.M.'s capability of handling novel metaphors was particularly challenging as the child came to consultation with a very assertive and rigid approach to language use. The originality of the treatment was to apply a multi-step explicit linguistic procedure. At first, a metaphor that could appear as an absurdity (X *is* Y) was transformed into a more acceptable sentence (X *is like* Y); then, the semantic features likely to provide a common ground between the two terms of the metaphor were identified, and on these grounds, a plausible interpretation was constructed.

After this experimental training, the child started handling the items of the Jnr MCT in a completely different manner. Although these items had nothing in common with the linguistic materials of the treatment, M.M.'s approach became at the same time more flexible (e.g., both the “rainbow” and the “milky way” could be conceived as “colorful parts of the sky”) and more consistent in his explanations.

It might be argued that, after four administrations of the same test, the child had learnt the acceptable answers. However, various evidences suggest that M.M.'s improvements with novel metaphors comprehension cannot be interpreted as a result of test repetition. First, the child never received a feedback on what was a good performance at the test, so he could not know whether his answers had improved or not. Second, what the test assesses is not the number of right answers, but the number of *well grounded explanations* of metaphors. Third, M.M. remarkably improved also in a subtest that was administered in the diagnostic phase and at the follow-up but not in the intermediate testing phases (APL-Medea; Lorusso, 2007). As the items of this subtest are mostly idioms, not expressed in the “X is Y” form used in the treatment, we may argue that our experimental training generated a “near transfer effect” which enhanced the general capability of handling metaphors. Thus, our study, in line with the results of previous research (Mashal and Kasirer, 2011; Persicke et al., 2012) indicates that it is possible to teach explicit procedures to enhance metaphor comprehension in children with ASD. In addition, the effectiveness of the experimental treatment, compared to the ineffectiveness of the training based on Theory of Mind suggests that the key factors to metaphor comprehension in this type of children (Norbury, 2005) are specifically linguistic and semantic. Actually, the child was trained to analyze both semantic similarities and differences between meanings pertaining to completely distinct semantic areas (e.g., sky and scarf)

We believe our findings contribute to the theoretical debate about novel metaphors comprehension in children with ASD.

We also speculate that a training centered upon the interactive construction of thinking maps between adult and child would enhance children's capability of producing semantic explanations of novel metaphors even at a younger age. This could be particularly helpful with children having weaknesses in semantic representations.

However, we must indicate at least three limitations of this study. First, the transfer effects of our training need to be explored by assessing comprehension of other types of figurative language. Second, there was no control group, and third, we had no feedback on whether M.M.'s improved capabilities adequately transferred to metaphor comprehension in everyday life conversation. These issues and the solidity of our training effects should be investigated in future research.

## Ethics statement

The Head of the Dipartimento di Pediatria e Neuropsichiatria Infantile (Un di Roma “Sapienza”) (Department of Pediatrics and Child Neuropsychiatry, Un of Rome “Sapienza”), where the study described in this article has been conducted provided the ethical approval. The ethical approval was obtained after the parents of the treated child gave their written, informed consent to the assessment and treatment of their child. The parents of the treated child have been informed throughout the whole process of assessment and treatment.

## Author contributions

SM is responsible for the whole empirical study. He wrote a first draft of the whole manuscript. MP and MO were interlocutors at every stage of the empirical study and the writing and revising process.

### Conflict of interest statement

The authors declare that the research was conducted in the absence of any commercial or financial relationships that could be construed as a potential conflict of interest.

## Footnotes

^1^At the time of this study, WISC III was the only version of the Wechsler scales available in Italy.

^2^A psychologist trained to administer the MCT and the APL Medea was responsible for the administration and blind to all the issues concerning the intervention.

^3^The APL subtest on figurative language includes 4 items for “verbal metaphors” and 4 items for “visual metaphors.” Despite the label “metaphor,” the test items mostly consist of idioms. The percentages of correct answers of Figure 1A are computed both in each subpart and in the total (8 items).

^4^The verbal items are given a score of 1 if the answer is metaphorical but not fully elaborate, and a score of 2 if it provides a more elaborate explanation. For instance,for the Italian item “Mario ha sempre la luna storta” (English equivalent: “Mario is always in a bad mood”), if the answer is “Mario reacts badly”, it is scored 1. If the answer is “Mario is always irritated”, it is scored 2. From the initial diagnosis to the follow-up assessment M.M. moved from 0 to 1 correct answer for visual metaphors and from one level 1 score to two level 2 scores for verbal metaphors.
